# Genetic and non-genetic determinants of thymic epithelial cell number and function

**DOI:** 10.1038/s41598-017-10746-8

**Published:** 2017-09-04

**Authors:** Daisuke Nagakubo, Brigitte Krauth, Thomas Boehm

**Affiliations:** 1 Department of Developmental Immunology, Max Planck Institute of Immunobiology and Epigenetics Stuebeweg 51, D-79108 Freiburg, Germany; 2 Present Address: Department of Fundamental Biosciences, Shiga University of Medical Science, Otsu, Shiga, 520-2192 Japan

## Abstract

The thymus is the site of T cell development in vertebrates. In general, the output of T cells is determined by the number of thymic epithelial cells (TECs) and their relative thymopoietic activity. Here, we show that the thymopoietic activity of TECs differs dramatically between individual mouse strains. Moreover, in males of some strains, TECs perform better on a per cell basis than their counterparts in females; in other strains, this situation is reversed. Genetic crosses indicate that TEC numbers and thymopoietic capacity are independently controlled. Long-term analysis of functional parameters of TECs after castration provides evidence that the number of *Foxn1*-expressing TECs directly correlates with thymopoietic activity. Our study highlights potential complications that can arise when comparing parameters of TEC biology across different genetic backgrounds; these could affect the interpretation of the outcomes of interventions aimed at modulating thymic activity in genetically diverse populations, such as humans.

## Introduction

The thymus is the site of T cell development in all vertebrates^1^; the thymic rudiment emerges from the pharyngeal endoderm in close apposition with neural crest-derived mesenchyme and non-crest mesoderm^2^, thus representing the primary lymphoid organ with the longest evolutionary history of spatial and functional conservation^3^. The thymic microenvironment attracts haematopoietic progenitors, specifies them to the T cell lineage, and orchestrates a complex series of events that culminates in the generation of a diverse population of mature T cells clonally expressing a self-tolerant repertoire of T cell receptors^4^. It has long been known that lymphopoietic activity in the thymus depends on an intact stromal compartment. This is most clearly evident from the observation that an alymphoid thymus is observed in mammals carrying mutations that inactivate the *Foxn1* gene^5^, which encodes a transcription factor of the forkhead family expressed in the thymic epithelium^6^. A peculiar feature of the thymus is the fact that it grows rapidly during early embryonic and adolescent stages, but then slowly involutes^7^, so that in aged animals only residual thymopoietic areas remain in this tissue^8^. The phenomenon of thymic involution has been associated with a low output of naive T cells and relative immunodeficiency in old age (reviewed in ref. 9). It is therefore conceivable that changes over time in the size and functionality of thymic epithelial cells not only determine the life history of the thymus but also the immune status of the individual, particularly in old age. With respect to the dynamics of the thymic epithelium, previous studies have indicated that the number of epithelial cells corresponds to the overall growth pattern of the thymus, with peak thymopoietic activity (expressed as the ratio of haematopoietic cells per thymic epithelial cells) at around 4 weeks of age^10^.

Studies in rodents have indicated that the size of the thymus varies between different strains of rats^11^ and mice^12^, but comparative studies focusing on their respective thymopoietic activity have not been carried out. The question remains as to whether the size of the thymus is associated with an overall larger number of TECs or with an increased activity per thymic epithelial cell. Moreover, no systematic analysis of potential strain-specific differences in TEC dynamics of male and female mice has yet been carried out. However, this type of information is crucial, as strain- and sex-specific differences in TEC number and function directly impact the interpretation of phenotypes resulting from perturbations of thymus function. For instance, more than 100 years ago, it was noted that castration of male rodents resulted in significant changes in thymus mass^13^. This observation has since been extended in numerous studies (for recent review, see ref. 14) and is even considered as a possible therapeutic strategy to reverse age-related thymic involution (for recent review, see ref. 15). Despite the fact that castration causes a rapid increase in thymic cellularity (for instance, see refs 16–18), its long-term effects on this parameter have not been studied in great detail. With respect to the effects of castration on the thymic stroma, it was established^10, 19^ that the increase in TEC numbers after castration is due to the expansion of mature mTECs, rather than of cTEC-like cells (now known to contain TEC progenitor cells^20^); although it is conceivable that the proliferative burst of TECs is short-lived, long-term follow-up studies have not been carried out. The short-term increase in thymus cellularity after sex steroid ablation was linked to an increase in the Ccl25 chemokine^19^, a key attractant for early thymic progenitors^21–23^, and also to the modulation of expression of Dll4^24^, the crucial Notch ligand responsible for T cell specification^25–27^.

The experiments presented in this report were designed to address some of the unresolved issues in thymus biology. Our results provide clear evidence for strain- and sex-specific differences in thymus function; this complex interplay makes it difficult to interpret the outcome of therapeutic interventions aimed at improving thymus function in situations of immunodeficiency or during physiological age-related involution.

## Results and Discussion

### Strain-specific differences

**Figure 1:**
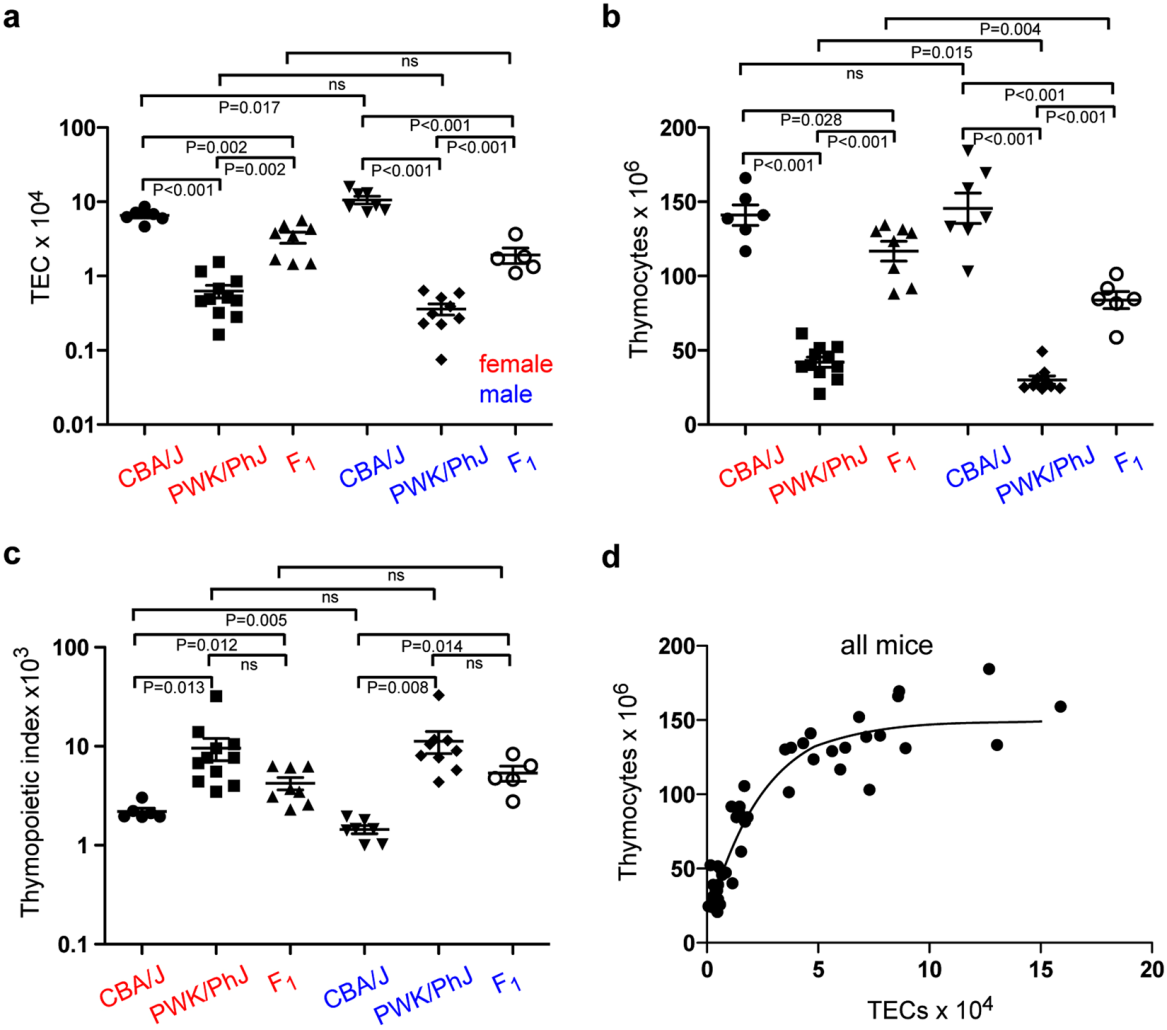
Functional characteristics of thymopoiesis in different mouse strains. (**a**) Number of thymic epithelial cells (TECs) in mice at 10 weeks of age. F_1_ denotes a hybrid between CBA/J and PWK/PhJ. (**b**) Number of CD45^+^ thymocytes. (**c**) Thymopoietic indices, calculated as dimensionless thymocyte/TEC ratios. (**d**) Non-linear relationship between thymocyte and TEC numbers; all mice were pooled for this analysis. In (**a**) to (**c**), means ± s.e.m.

Previous studies indicated that the weight ratio of thymus to whole body differs among different mouse strains and between the sexes^12^. Here, we focus on two mouse strains, CBA/J and PWK/PhJ, which were shown to differ both in relative thymus weight and in the male and female ratio of thymus weight (http://phenome.jax.org/db/qp?rtn=views/measplot&brieflook=10416&projhint=Deschepper1). We examined whether the number of epithelial cells in the thymic microenvironment was correlated with thymus weight; thymus weight is largely determined by the number of haematopoietic cells which outnumber stromal cells by two to three orders of magnitude^2^. We found that the number of thymic epithelial cells (TECs) varies greatly between strains. For instance, at 10 weeks of age, the thymus of males of the CBA/J strain is composed of 10.61 ± 1.23 × 10^4^ (mean ± s.e.m.; n = 6) thymic epithelial cells (TECs), while the thymi of males of the PWK/PhJ strain harbour a mere 0.33 ± 0.12 × 10^4^ TECs (mean ± s.e.m.; n = 11) (Fig. 1a), resulting in a 30-fold difference in TEC numbers per thymus. In female mice, the difference in TEC numbers is approximately 13-fold (6.58 ± 0.54 × 10^4^ TECs [mean ± s.e.m.; n = 7] in CBA/J, and 0.36 ± 0.06 × 10^4^ TECs [mean ± s.e.m.; n = 9] in PWK/PhJ). Of note, the numbers of TECs also differ between male and female mice of one strain. Whereas male CBA/J mice exhibit higher numbers of TECs per thymus than females, the situation is reversed in male PWK/PhJ mice (Fig. 1a).

The numbers of thymocytes in the CBA/J and PWK/PhJ strains also vary, in general agreement with the variations in TEC numbers (Fig. 1b). However, when expressed as the numbers of thymocytes per numbers of TECs (a measure we refer to here as the thymopoietic index), striking differences between strains and sex become evident. In general, the lymphopoietic activity of TECs of the PWK/PhJ strain is several-fold higher than that of CBA/J mice; moreover, whereas male CBA/J mice exhibit an overall lower thymopoietic activity than their female counterparts, the situation is reversed in PWK/PhJ mice (Fig. 1c). Note that the thymopoietic index represents an average value for the epithelial compartment as a whole, and hence does not take into account possible differential lymphopoietic activities of different types of TECs. Since our isolation protocol for stromal cells results in complete dissociation of thymic tissue, the observed differences in thymocyte and TEC numbers suggest that thymus size is controlled by genetic background and sex.

In order to examine the possible genetic basis of these phenomena, we generated F_1_ hybrids between CBA/J and PWK/PhJ strains. The results shown in Fig. 1a indicate that, compared to the parent strains, F_1_ males and females exhibit intermediate numbers of TECs, suggesting co-dominant contributions of factor(s) regulating the size of the TEC compartment. Interestingly, however, the sex-specific differences between male and female mice with respect to total TEC numbers phenocopy the PWK/PhJ characteristics; F_1_ hybrids exhibit a higher number of TECs in females than in males; the ratios of the numbers of TECs in females and males are 0.62 for CBA/J, 1.75 for PWK/PhJ and 1.73 for (CBA/J x PWK/PhJ)F_1_ hybrids (Fig. 1a). This indicates that the underlying genetic determinant(s) of CBA/J regulating these characteristics are recessive when combined with those of PWK/PhJ. With respect to thymocyte numbers and thymopoietic indices, F_1_ hybrid mice again exhibit intermediate values (Fig. 1b,c). Of note, the male/female ratios of thymopoietic activities again reflect the PWK/PhJ phenotype (Fig. 1c). We note that the commonly used C57BL/6 strain exhibits thymopoietic characteristics similar to CBA/J mice (Supplementary Fig. 1).

Collectively, these data indicate a non-linear relationship between the number of TECs and the number of thymocytes (Fig. 1d), supporting the notion that the strain- and sex-specific qualities rather than the numbers of TECs as such are the determining factors of overall thymopoietic activity in the thymus.

### Age-related changes

**Figure 2:**
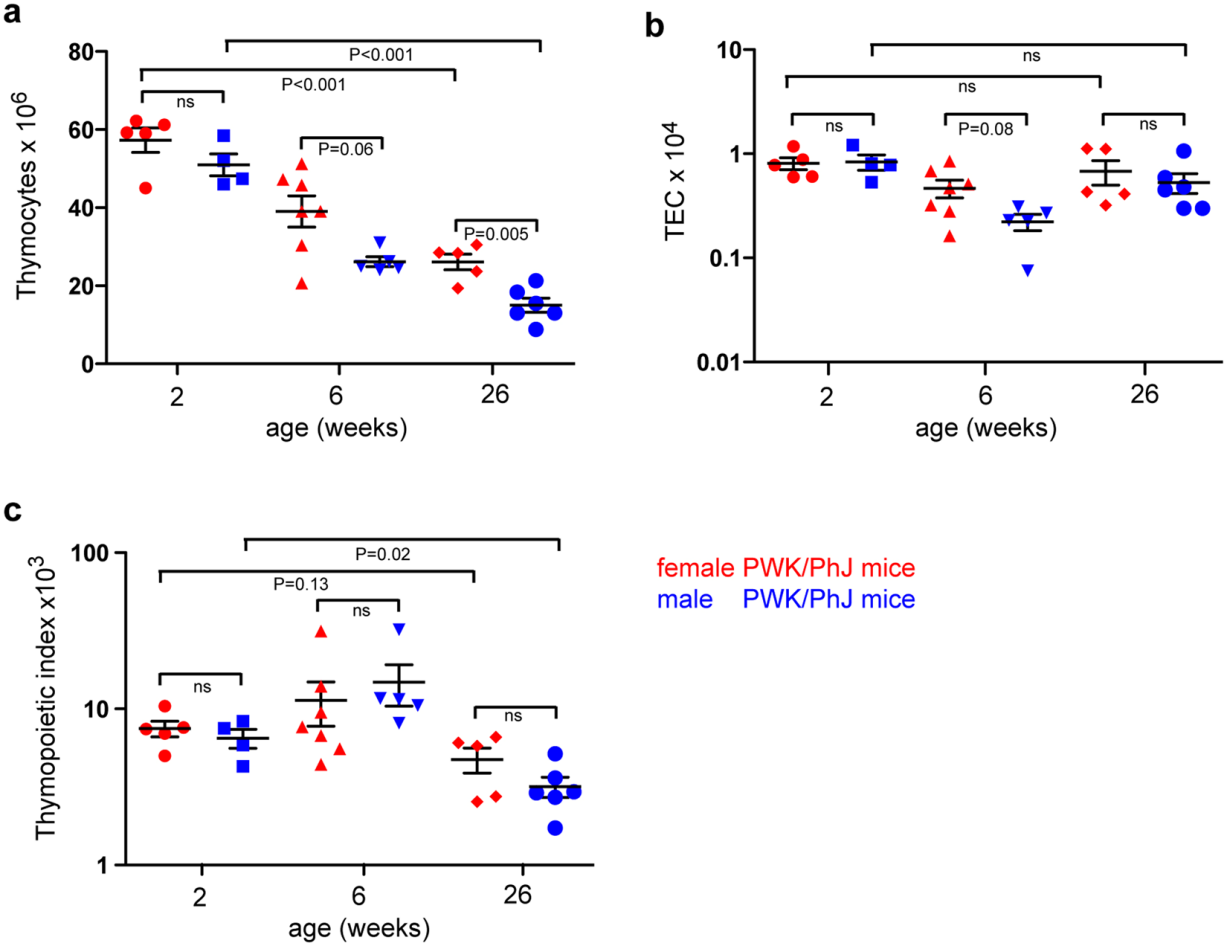
Time-dependent changes in thymopoietic characteristics in PWK/PhJ mice. **(a)** Number of CD45^+^ thymocytes. (**b**) Number of thymic epithelial cells (TECs). (**c**) Thymopoietic indices, calculated as thymocyte/TEC ratios. Means ± s.e.m.

Having established that both strain and sex determine the magnitude of the thymopoietic index, we investigated whether age also affects this parameter. In this regard, it is particularly interesting to examine this phenomenon in the PWK/PhJ strain, because of its unusual TEC phenotype. It is well known that thymopoietic activity declines with age^10, 18^, and this phenomenon is also evident in the PWK/PhJ strain. Compared to the numbers at two weeks of age, thymic cellularity of PWK/PhJ mice declines by a factor of 2-3 until six months of age, with males being disproportionally affected (Fig. 2a); by contrast, the number TECs decreases only moderately from adolescence to about 6 months of age (Fig. 2b). As a result, the thymopoietic index declines with age (Fig. 2c). A qualitatively similar phenomenon is observed in C57Bl/6 mice (Supplementary Fig. 1; see also ref. 28). In a morphometric study on human thymi, it was found that the maximum size of the thymus is reached at the age of about one year; hence this suggests that the involution of the epithelial component of the thymus is independent of puberty^29^. Indeed, this corresponds to the finding that within the thymic epithelial compartment, the number of *Foxn1*-expressing cells, the key supporters of thymopoietic activity^30, 31^, declines shortly after birth^18, 30^. Collectively, the observed age-related changes provide additional support for the notion that the quality (for instance, as reflected in the fraction of *Foxn1*-positive TECs; see below) rather than the quantity of TECs is the key factor determining thymopoietic capacity.

### Sex-specific differences

**Figure 3:**
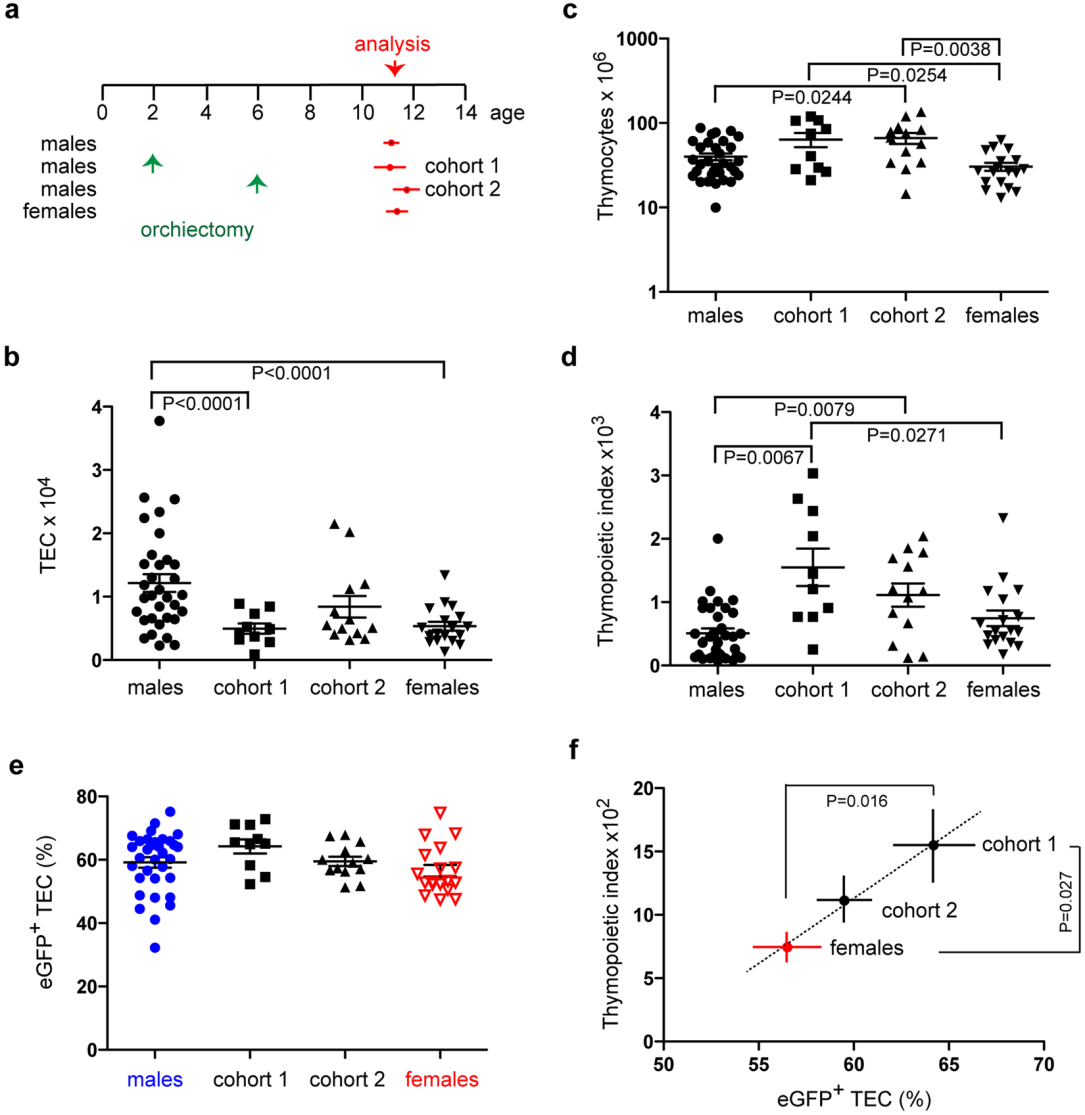
Long-term consequences of orchiectomy. (**a**) Two cohorts of male *Foxn1:eGFP* transgenic mice were castrated at either 2 or 6 months of age; their thymopoietic parameters were examined at approximately 12 months of age (the means and s.e.m. of analysis time points are indicated) and compared to those of a male control group (combined cohorts of untreated and sham-operated males, which did not differ in the parameters studied here), and of female mice. (**b**) Number of thymic epithelial cells (TECs). (**c**) Number of CD45^+^ thymocytes. (**d**) Thymopoietic indices, calculated as thymocyte/TEC ratios. (**e**) Fraction of eGFP^+^ cells as a measure of *Foxn1*-expression in the thymic epithelium. The total numbers (mean values) of eGFP^+^ TECs is as follows. Males: 7,200 cells; cohort 1: 3,200 cells; cohort 2: 5,000 cells; females: 3,000 cells. (**f**) Linear relationship between the thymopoietic indices and the fraction of eGFP^+^ TECs. Means ± s.e.m.

We sought to substantiate this conclusion by exploiting the observation that the thymopoietic index differs between males and females (Fig. 1; Supplementary Fig. 1). We were particularly interested in the potential role of androgens; to do so, we examined the effects of bilateral orchiectomy on TEC numbers and their thymopoietic activity. As shown schematically in Fig. 3a, we castrated male mice at 2 or 6 months of age and analysed them at about one year of age, that is 10 months (cohort 1) and 6 months (cohort 2) after orchiectomy. Untreated males and sham-operated males (which did not differ significantly from each other in the parameters investigated here) were used as a combined control group. In our strain of mice (a mixed C57Bl/6;FVB background transgenic for *Foxn1:eGFP* ^32^), 12 month-old control males exhibit about twice as many TECs as their female siblings (Fig. 3b). Despite this significant difference in TEC numbers, the thymi of both sexes support approximately an equal number of thymocytes (Fig. 3c); this results in a higher thymopoietic index for females than males (Fig. 3d), comparable to what is seen in CBA/J (Fig. 1) and C57BL/6 (Supplementary Fig. 1) mice. In the male control group, 59.2 ± 1.7% (mean ± s.e.m.) of TECs are eGFP^+^ (*Foxn1*-expressing) cells, associated with a thymopoietic index of 510 ± 76 (mean ± s.e.m.), whereas in female mice, 56.5 ± 1.8% (mean ± s.e.m.) of TECs are eGFP^+^ and associated with a thymopoietic index of 744 ± 124 (mean ± s.e.m.) (Fig. 3b,d). This suggests that each percent of eGFP^+^ TECs contributes to the thymopoietic index 8.6 points in males, and 13.2 points in females, amounting to a decrease of about one third in thymopoietic capacity in the presence of androgens.

Interestingly, males castrated at 2 months of age and analysed about 10 months later have approximately the same number of TECs as females of the same age, suggesting that in this strain of mice, androgens are required to sustain the larger TEC compartment: by contrast, males castrated at 6 months of age and analysed about 6 months later exhibit intermediate numbers of TECs, indicating that TEC numbers decline rather slowly following androgen withdrawal (Fig. 3b). This observation is in line with results from a recent study of C57BL/6 mice which reported lower numbers of TECs in castrated males than in their untreated counterparts^28^. Remarkably, the numbers of thymocytes in castrated males are higher than those of male controls and females (Fig. 3c), resulting in thymopoietic capacities that even exceed those of females (Fig. 3d). Thus, although androgen withdrawal reduces the number of TECs, the sudden change in the hormonal milieu is conducive to the generation of thymopoietically more active TECs.

Next, we studied the possible mechanism for this observation. We found that the higher thymopoietic capacity of TECs in castrated males is associated with a higher percentage of eGFP-positive *Foxn1*-expressing TECs (Fig. 3e). Interestingly, an increase in *Foxn1* expression levels was previously observed as early as 8 days after castration, although at that time point the number of *Foxn1*-positive cells had not yet changed^18^. It thus appears that the acute withdrawal of androgens results in rapid^18^ and sustained changes (Fig. 3d,f) in the composition and functional capacity of the thymic microenvironment.

As is evident from Fig. 3f, a linear relationship exists between the magnitude of the thymopoietic index and the percentage of eGFP^+^ (*Foxn1*-expressing) TECs, indicating that androgens affect the efficiency with which eGFP^+^ TECs support T cell development; *i.e*., eGFP^+^ TECs in castrated mice behave as if they were female eGFP^+^ TECs. Androgen withdrawal thus converts “male-type” eGFP^+^ TECs into their more active “female-type” counterparts, in addition to changing the overall composition of TECs with features of “younger” age^10, 18, 30^, representing a cellular correlate of the rejuvenation effect associated with of castration^14^.

More generally, the present observations provide another example for the notion that *Foxn1* expression is required for thymopoietic activity^30, 31^. It also suggests that even a seemingly minor increase in the fraction of *Foxn1*-positive TECs can have a profound effect on the number of developing thymocytes in the thymus.

## Conclusions

In conclusion, our study has illustrated a complex interplay between genetic and non-genetic factors regulating thymopoietic activity as physiologically relevant key aspects of the thymic microenvironment. Our results set the stage for the identification of the genetic factors underlying this striking strain-specific difference in thymopoietic phenotype. The present findings reinforce the notion that subtle changes in *Foxn1* expression can have dramatic effects on thymus function. Hence, future work will be aimed at examining the mechanisms that impact the expression of the key *Foxn1* transcription factor gene with the aim of achieving a rejuvenation effect without the deleterious consequences associated with androgen withdrawal or blockade.

From a practical perspective, our results illustrate the complications arising when comparing parameters of TEC biology across different genetic backgrounds. Thus, caution should be exercised when interpreting the outcomes of pharmacological or other interventions aimed at modulating thymic activity in genetically diverse populations such as humans.

## Methods

### Mice

PWK/PhJ mice (wild-derived, inbred strain) were obtained from Jackson Laboratories (stock number 003715). The strain is descended by sib mating from a single pair of mice of the subspecies *Mus musculus musculus* caught in 1974 in Lhotka, Czech Republic^33^. CBA/J mice (stock number 000656) were obtained from Jackson Laboratories. C57BL/6 mice are maintained in the Max Planck Institute of Immunobiology and Epigenetics. *Foxn1:eGFP* transgenic mice were described earlier^32^. Mice were kept in the animal facility of the Max Planck Institute of Immunobiology and Epigenetics under specific pathogen-free conditions. All animal experiments were performed in accordance with the relevant guidelines and regulations, approved by the review committee of the Max Planck Institute of Immunobiology and Epigenetics and the Regierungspräsidium Freiburg, Germany (licence AZ 35-9185.81/G-12/85).

### Flow cytometry

To generate single cell suspensions for TEC staining, thymi were finely minced with scissors, and then digested with a cocktail of collagenase type 4 (200 µg/mL), neutral protease (200 µg/mL) and DNaseI (500ng/mL) in RPMI 1640 + 2% FCS for up to 90 minutes at 37 °C with gentle agitation. Digestion was routinely carried out in a final volume of 1 ml per thymic lobe. Care was taken to keep the small tissue fragments afloat during the early phases of the digestion process to facilitate penetration of enzymes into the tissue; in some instances, the digestion is carried out in two sequential steps, removing the liberated cells from the supernatant after half the incubation time and adding fresh digestion buffer. Following digestion, EDTA was added to a final concentration of 2 mM, which facilitates the disaggregation of any remaining small cell clusters of epithelial cells presumably owing to the disruption of E-cadherin complexes. In this way, the entire thymic tissue could be dissociated into a single cell suspension, avoiding isolation/quantification artefacts associated with possible strain-dependent differences in the composition of the thymic microenvironment. Cells were then washed with RPMI 1640 + 2% FCS, and re-suspended in PBS supplemented with 0.5% BSA for staining. Cell surface staining (see Supplementary Table 1 for antibodies) was performed at 4 °C in PBS supplemented with 0.5% BSA and 0.02% NaN_3_. Thymic epithelial cells have the surface phenotype CD45^−^/EpCAM^+^; thymocytes are CD45^+^/EpCAM^−^. Note that the enzymatic cocktail required to liberate thymic epithelial cells destroys the extracellular domains of CD4 and CD8 surface markers (but not that of the CD45 molecule); hence, when analysis of thymocyte subsets is required, thymocyte suspensions must be prepared in parallel by mechanical liberation, best achieved by gently pressing thymic lobes through 40 µm sieves. For quantitative analysis, consideration must be given to the fact that left and right thymic lobes are of different size (the left lobe being invariably smaller).

For the calculation of the thymopoietic index, the number of CD45^+^/EpCAM^−^ cells was divided by the number of CD45^−^/EpCAM^+^; the index is equivalent to the inverse of the fraction of CD45^−^/EpCAM^+^ cells in the flow cytometric profile (for instance, a fraction of 0.1% of CD45^−^/EpCAM^+^ cells in a preparation is equivalent to a thymopoietic index of 1,000).

The fraction of *Foxn1*-expressing cells was determined by eGFP-fluorescence emanating from the *Foxn1:eGFP* transgene^32^, which faithfully recapitulates the acute levels of *Foxn1* expression^18^.

### Orchiectomy

This procedure was carried out essentially as described in ref. 34. Sham-operated males underwent all steps except that after mobilization of the testicles, they were repositioned to their original location. For the parameters examined here, no differences were observed for untreated males and sham-operated males; hence the results of these groups were pooled for subsequent analysis.

### Statistical analysis

t-tests (two-tailed) were used to determine the significance levels of the differences between the means of two independent samples, considering equal or unequal variances as determined by the F-test. For multiple tests, the conservative Bonferroni correction was applied.

## Electronic supplementary material

Supplementary Information
