# Supplementary material for: Genetic and non-genetic determinants of thymic epithelial cell number and function
Source: Sci Rep. 2017 Sep 4;7:10314. doi: 10.1038/s41598-017-10746-8 (PMC5583284; doi:10.1038/s41598-017-10746-8)
Supplement: Supplementary file 1 — Supplementary Information [file 41598_2017_10746_MOESM1_ESM.pdf]

Supplementary Information for

Genetic and non-genetic determinants of thymic epithelial cell number and function

Daisuke Nagakubo, Brigitte Krauth and Thomas Boehm

Supplementary Figure 1

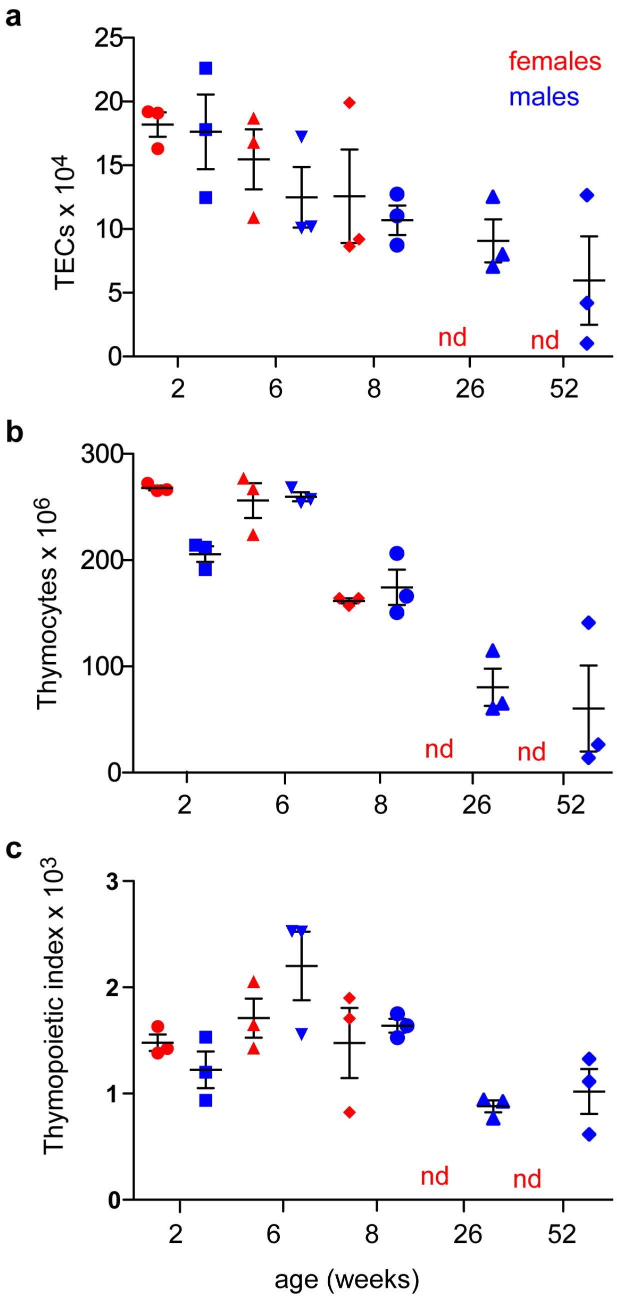

Supplementary Figure 1. Time-dependent changes in thymopoietic characteristics in C57BL/6 mice. (a) Number of thymic epithelial cells (TECs). (**b**) Number of $CD45^+$ thymocytes. (**c**) Thymopoietic indices, calculated as thymocyte/TEC ratios.

Results for female mice are shown in red, those for male mice in blue; means±s.e.m.

Supplementary Table 1. Antibodies and staining reagents for flow cytometry

**Antigen/Reagent**	**Clone**	**Conjugate**	**Supplier**
CD3	145-2C11	APC	eBioscience
CD4	GK1.5	FITC	BioLegend
CD8a	53-6.7	PE	eBioscience
CD19	1D3	PE Cy7	eBioscience
CD44	IM7	PE	BD Bioscience
CD45	30-F11	PE Cy7	BioLegend
CD62L	MEL-14	FITC	eBioscience
EpCAM	G8.8	APC	BioLegend
Ly51	6C3	PE	eBioscience
mouse IgG1	goat polyclonal	biotin	SouthernBiotech
mouse IgG (H+L)	rat polyclonal	FITC	Jackson ImmunoResearch
rabbit IgG (H+L)	goat polyclonal	Alexa Fluor 488	Invitrogen
rat IgG (H+L)	donkey polyclonal	Cy3	Jackson ImmunoResearch
Streptavidin	-	Cy3, Cy5	Jackson ImmunoResearch
Streptavidin	-	FITC, PE or Alexa Fluor v450	eBioscience
UEA-1	-	biotin, FITC	Vector Laboratories
